# Inhaled Isopropyl Alcohol for Refractory Cannabis Hyperemesis Syndrome: A Case With Pneumomediastinum and Pneumothorax

**DOI:** 10.7759/cureus.93493

**Published:** 2025-09-29

**Authors:** Abdul Rehman, Muhammad Ahmad Javed

**Affiliations:** 1 Emergency Department, St. Luke's General Hospital, Kilkenny, IRL

**Keywords:** boerhaave syndrome, cannabis hyperemesis syndrome, conservative management, isopropyl alcohol, pneumomediastinum, refractory vomiting, spontaneous tracheal rupture, subcutaneous emphysema, vomiting-induced injury

## Abstract

Cannabis hyperemesis syndrome (CHS) is a well-recognized condition associated with chronic cannabis use, characterized by recurrent nausea, retching, vomiting, and relief with hot showers. Management is challenging, particularly as symptoms are refractory to conventional antiemetic therapy. We report the case of a 26-year-old man with a history of daily cannabis use who presented with severe retching and vomiting, complicated by pneumothorax and pneumomediastinum. His nausea and retching persisted despite multiple antiemetic therapies but responded rapidly to inhaled isopropyl alcohol (IPA) swabs. This case highlights the potential role of non-pharmacological aromatherapy as a safe and effective adjunct in managing refractory CHS while also underscoring the serious complications that can occur from severe persistent emesis.

## Introduction

Cannabis hyperemesis syndrome (CHS) is a paradoxical adverse effect of chronic cannabis use, first described in 2004 [1]. Patients typically present with cyclical vomiting, abdominal pain, and the characteristic behavior of compulsive hot bathing for symptomatic relief [2]. While the pathophysiology remains unclear, proposed mechanisms include the dysregulation of the endocannabinoid system and delayed gastric emptying [3]. Complications of severe and protracted emesis may include dehydration, electrolyte disturbances, acute kidney injury, and, rarely, life-threatening conditions such as esophageal perforation [4]. Treatment options are limited, with haloperidol, benzodiazepines, topical capsaicin, and supportive care being commonly employed [5]. Recent studies suggest that inhaled isopropyl alcohol (IPA) may offer a rapid, non-pharmacological alternative for acute nausea and vomiting [6]. Sustained cannabis cessation remains the only definitive therapy and is associated with the complete remission of CHS symptoms.

We present a case of CHS complicated by pneumomediastinum and bilateral pneumothorax, in which inhaled isopropyl alcohol provided effective relief of persistent nausea and retching when pharmacological agents failed.

## Case presentation

A 26-year-old man with a history of gastroesophageal reflux disease and cannabis addiction presented to the emergency department (ED) with central chest pain, epigastric pain, chills, and decreased oral intake for the past 12 hours. He described a five-day history of persistent nausea, retching, and non-bilious vomiting exceeding 10 episodes per day, associated with lethargy, lightheadedness, and reduced urine output. His partner reported noticing him taking frequent hot showers over the past three days. He denied hematemesis, cough, shortness of breath, recent travel, allergies, or the initiation of any new medications. His past medical history revealed multiple hospital admissions over the prior four months for similar unexplained vomiting episodes. On further questioning, he disclosed daily cannabis use for the last five years, with an escalated use over the last six months. He denied alcohol, tobacco, or other recreational substances.

On examination, he appeared distressed from retching. His vital signs revealed hypotension (blood pressure: 86/55 mmHg), tachycardia (heart rate: 128 beats per minute {bpm}), a respiratory rate of 20 breaths per minute, a temperature of 37.4°C, and an oxygen saturation of 96% on room air. He was lethargic and pale-looking, with dry mucous membranes and poor skin turgor. Abdominal examination showed mild epigastric tenderness without any signs of peritonitis. Chest examination revealed equal air entry bilaterally and palpable subcutaneous emphysema extending into the neck. Neurological findings were normal.

**Figure 1:**
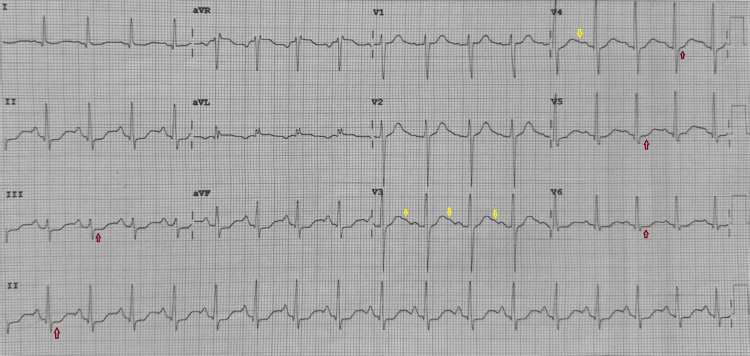
ECG showing sinus tachycardia along with wide-spread ST segment depression (red arrows) and the presence of U waves (yellow arrows). ECG: electrocardiogram

**Figure 2:**
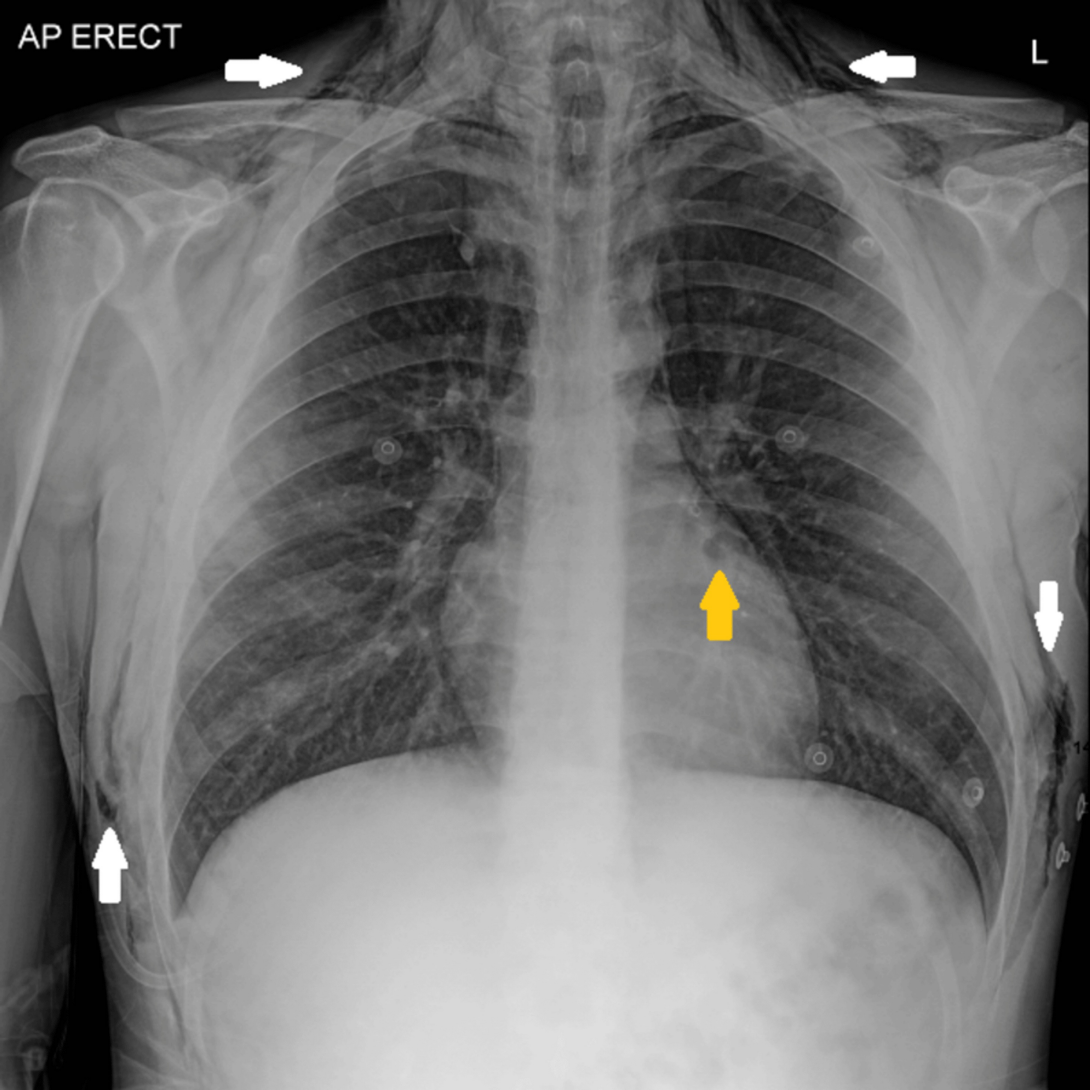
X-ray of the chest demonstrating extensive surgical emphysema (white arrows) in the chest wall and subcutaneous tissues, extending up to the neck on both sides, and associated pneumomediastinum (yellow arrow).

Laboratory investigations revealed hypokalemia with metabolic alkalosis, elevated lactate, and abnormal urea and creatinine levels (Tables 1, 2). The electrocardiogram (ECG) showed sinus tachycardia with U waves and ST depression, consistent with hypokalemia, but without QT prolongation (Figure 1). Chest radiography demonstrated pneumomediastinum and subcutaneous emphysema without free intra-abdominal air (Figure 2).

**Table 1:** Laboratory investigations. PT, prothrombin time; APTT, activated partial thromboplastin time; INR, international normalized ratio; MCV, mean corpuscular volume; WBC, white blood cell; RBC, red blood cell; ALT, alanine transaminase; AST, aspartate transaminase; CRP, C-reactive protein; eGFR, estimated glomerular filtration rate

Test	Result	Normal Range
WBC count	16 × 10^9^/L	4-10 × 10^9^/L
RBC count	5.5 × 10^12^/L	4.5-5.5 × 10^12^/L
Hemoglobin	19.0 g/dL	13-17 g/dL
Platelets	275 × 10^9^/L	150-450 × 10^9^/L
MCV	87.9 fL	83-101 fL
CRP	114 mg/L	<5 mg/L
Serum sodium	132 mmol/L	135-145 mmol/L
Serum potassium	2.2 mmol/L	3.5-5.0 mmol/L
Chloride	79 mmol/L	98-107 mmol/L
Bicarbonate	35 mmol/L	22-28 mmol/L
Urea	8.2 mmol/L	2.5-7.8 mmol/L
Creatinine	373 mmol/L	45-106 μmol/L
eGFR	28.2 mL/minute/1.73 m^2^	>90 mL/minute/1.73 m^2^
AST	30 U/L	15-50 U/L
ALT	21 U/L	5-55 U/L
Total bilirubin	19.8 μmol/L	2.0-21.0 μmol/L
Amylase	71 U/L	28-100 U/L
Albumin	62 g/L	35-50 g/L
PT	10.4 seconds	9.4-12.5 seconds
INR	0.9 seconds	0.8-1.2 seconds
APTT	30.1 seconds	25-40 seconds

**Table 2:** Venous blood gas. pCO_2_, partial pressure of carbon dioxide; pO_2_, partial pressure of oxygen

Test	Result	Units	Normal Range
pH	7.51		7.32-7.43
pCO_2_	5.9	kPa	4.6-6.4
pO_2_	5.1	kPa	2.3-5.5
Sodium	132	mmol/L	136-145
Potassium	2.2	mmol/L	3.5-5.1
Chloride	79	mmol/L	98-107
Ionized calcium	1.08	mmol/L	1.16-1.32
Glucose	15.8	mmol/L	3.6-5.3
Lactate	5.7	mmol/L	0.9-1.7
Hematocrit	60	%	35-51
Total hemoglobin	19.0	g/dL	11.7-17.4
Base excess	10.0	mmol/L	-2.0-3.0
Anion gap	18	mmol/L	10-20
Bicarb, standard	31.5	mmol/L	22.0-29.0
Bicarb, actual	35.0	mmol/L	22.0-29.0

**Figure 3:**
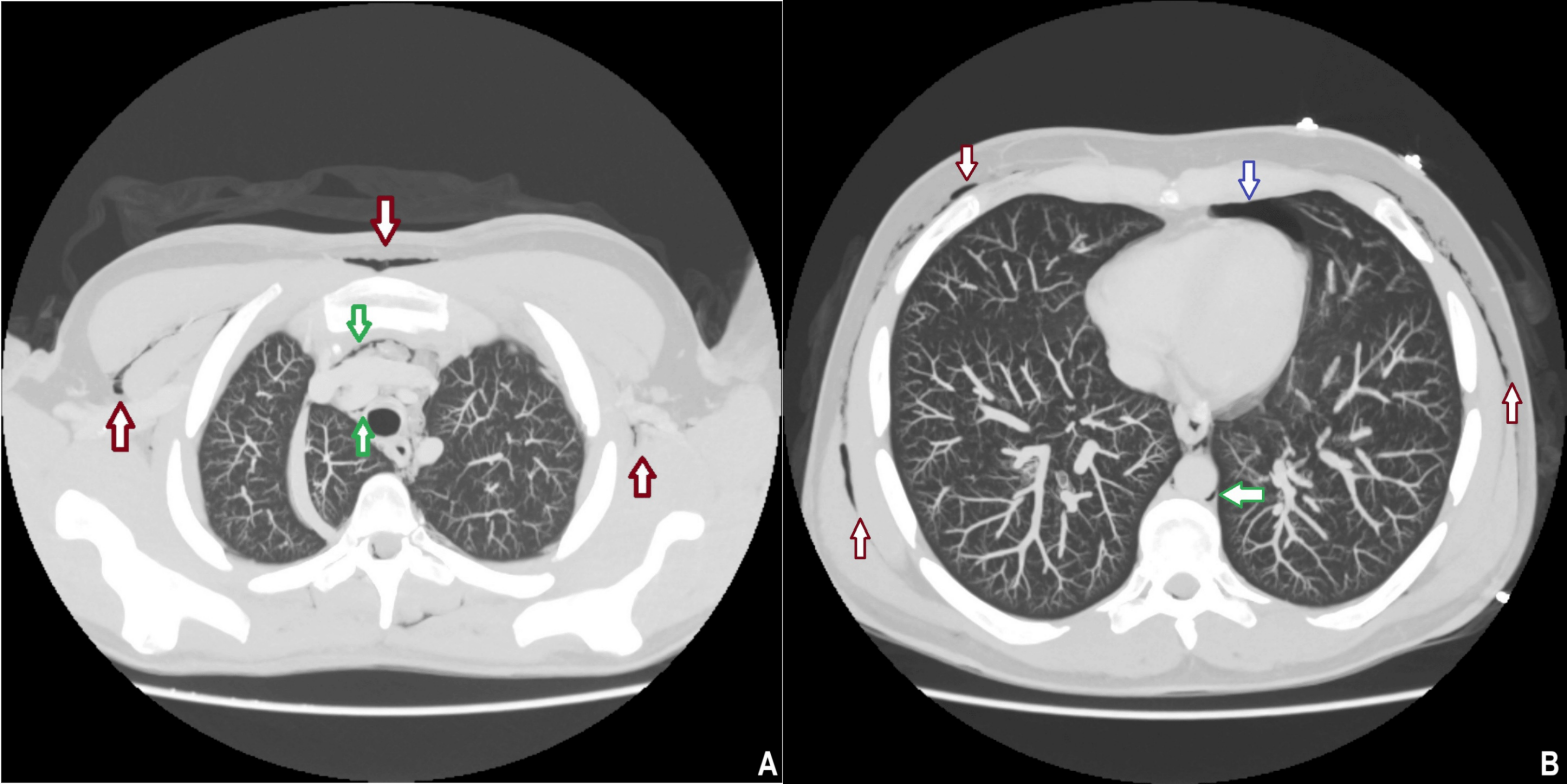
Axial CT of the thorax demonstrating extensive surgical emphysema (red arrows) in the chest wall, associated left-sided pneumothorax (blue arrow), and pneumomediastinum (green arrows) with air outlining the mediastinal structures. CT: computed tomography

**Figure 4:**
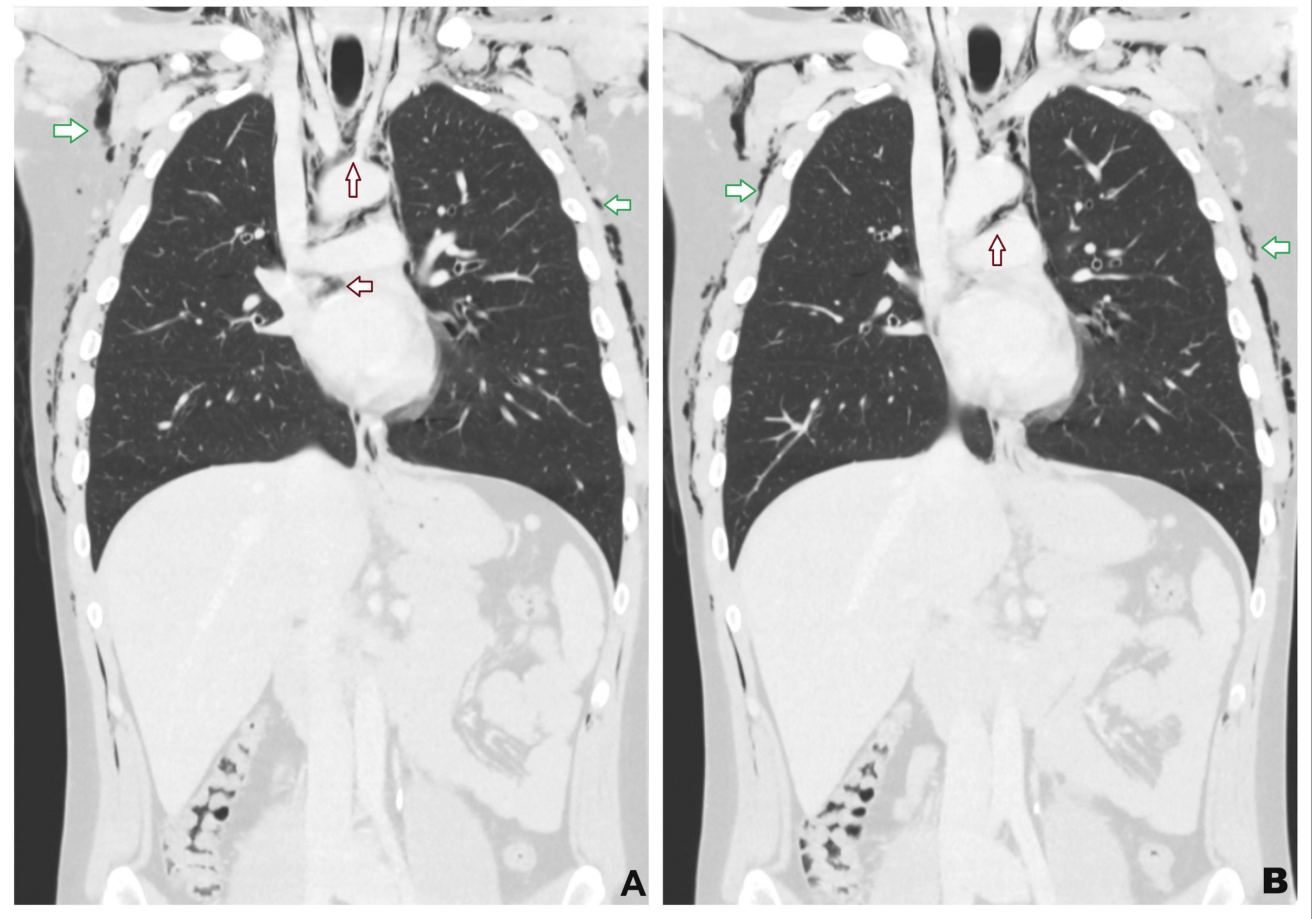
Coronal CT of the thorax demonstrating extensive surgical emphysema within the chest wall and subcutaneous tissues (green arrows). There is also an associated pneumomediastinum, with air outlining the mediastinal structures (red arrows). CT: computed tomography

**Figure 5:**
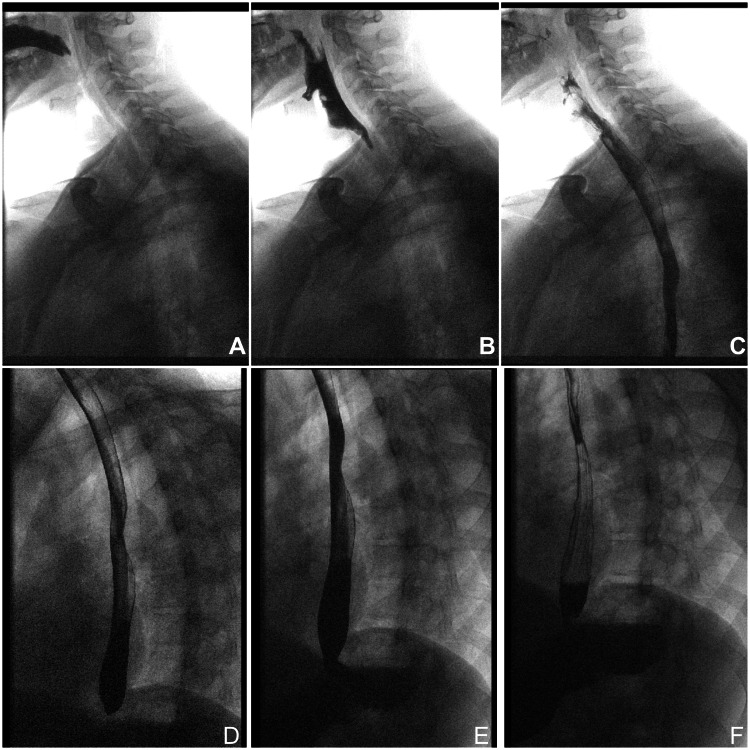
Contrast esophagogram demonstrating a normal esophagus, with no evidence of perforation or contrast extravasation.

The diagnosis of CHS and Boerhaave syndrome was suspected based on clinical history and cannabis use. Initial management included intravenous (IV) fluids, intravenous analgesia, ondansetron, esomeprazole, cyclizine, and intramuscular haloperidol with minimal relief. Given the initial imaging findings, an urgent computed tomography (CT) scan of the thorax, abdomen, and pelvis was obtained, revealing pneumomediastinum, bilateral pneumothorax, and extensive surgical emphysema extending up to the neck without any evidence of pleural effusion or localized collection (Figures 3, 4). A contrast esophagogram, using water-soluble contrast (Omnipaque), subsequently followed by barium contrast, was obtained and was negative for any esophageal leak or perforation (Figure 5). Upper gastrointestinal and cardiothoracic surgical teams were consulted, and they recommended conservative management with nil per os, total parenteral nutrition, high-flow oxygen, broad-spectrum intravenous antibiotics, analgesia, and close monitoring in the intensive care unit. Topical capsaicin cream (0.1%) was also trialed without any symptomatic improvement.

**Figure 6:**
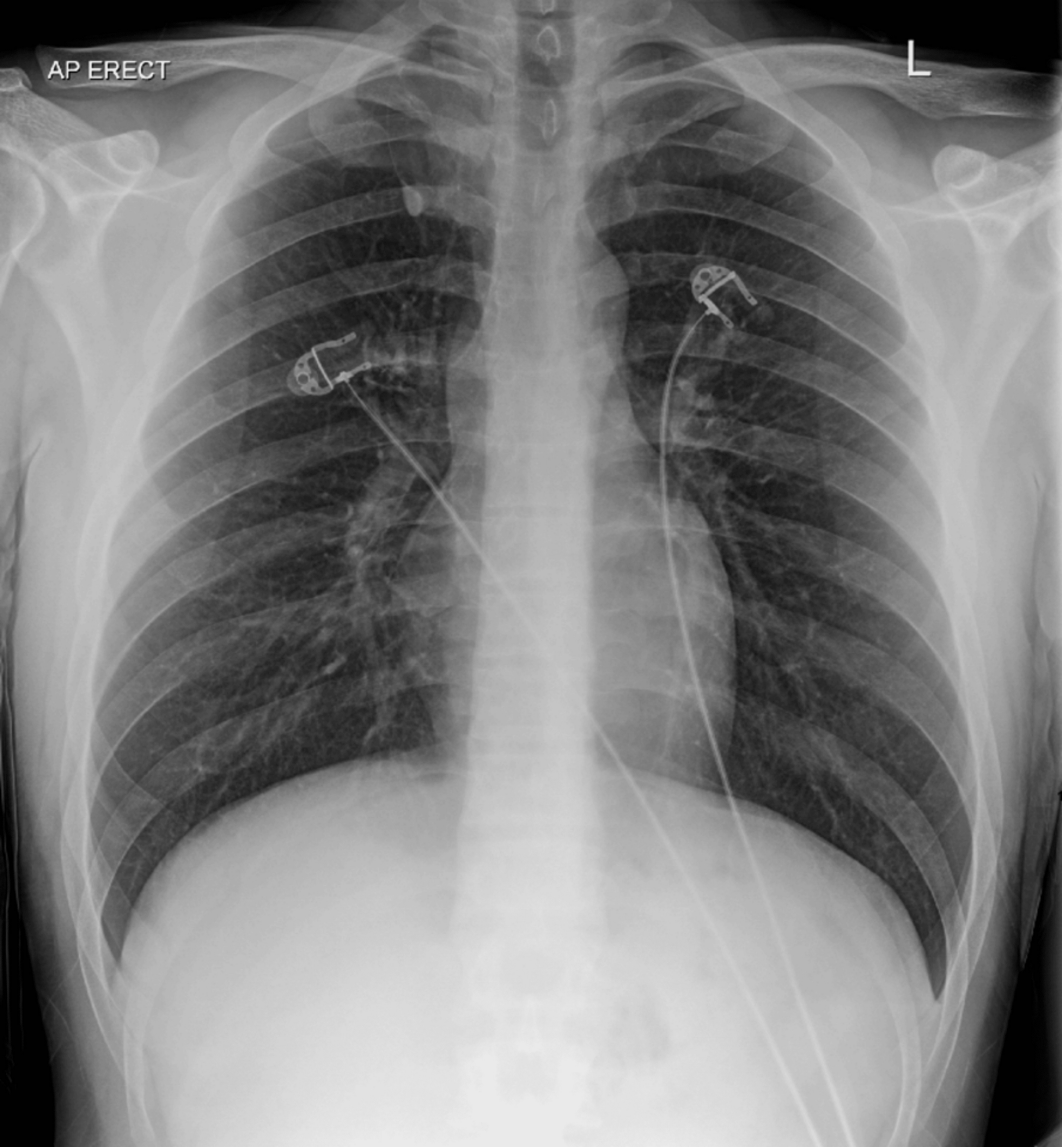
Follow-up chest X-ray showing the resolution of surgical emphysema or pneumomediastinum.

Despite these measures, nausea and retching persisted on day 2. The gastroenterology team recommended non-pharmacological aromatherapy with inhaled isopropyl alcohol swabs, which resulted in rapid symptomatic relief. With continued fluid resuscitation and electrolyte correction, his renal function and metabolic abnormalities normalized. Serial chest imaging demonstrated gradual resolution of pneumothorax and pneumomediastinum. Oral fluids were reintroduced on day 9 and were well-tolerated. He was discharged on day 14, following counseling regarding cannabis cessation. A follow-up X-ray and assessment at four weeks after discharge showed no evidence of subcutaneous emphysema or pneumomediastinum, and he reported recovering well with no complaints (Figure 6).

## Discussion

Cannabis hyperemesis syndrome is increasingly encountered in emergency and inpatient settings as community cannabis potency and use rise. It is conceptualized as a disorder of gut-brain interaction with a characteristic triphasic course (prodromal, hyperemetic, and recovery) and stereotyped hot‑water bathing behavior [1,2]. Contemporary guidance from the American Gastroenterological Association (AGA) emphasizes a positive clinical diagnosis using Rome IV-aligned criteria, recurrent stereotyped emesis in the context of chronic cannabis exposure with improvement on abstinence, after the reasonable exclusion of alternative etiologies [1,3]. Our patient's long-term history of heavy daily use, cyclical hospital presentations, hot showering, and resolution with supportive care and cessation counseling are concordant with these criteria.

Prolonged retching in CHS can precipitate profound dehydration, electrolyte derangements (notably hypokalemia), acute kidney injury, and barotrauma [4]. Spontaneous pneumomediastinum and pneumothorax may occur via the Macklin effect, and, more rarely, transmural esophageal rupture (Boerhaave syndrome) may complicate severe episodes, mandating urgent cross-sectional imaging and multidisciplinary input [4-6]. Our patient's CT‑confirmed esophageal perforation with concomitant pneumomediastinum and pneumothorax underscores the need for early recognition, high‑flow oxygen, broad-spectrum antimicrobials, nil per os with parenteral nutrition, and close cardiopulmonary monitoring, consistent with established management principles for Boerhaave syndrome [7].

Haloperidol, benzodiazepines, and topical capsaicin are commonly used when first-line 5-hydroxytryptamine type 3 (5-HT3) antagonists fail. A randomized trial demonstrated haloperidol's superiority over ondansetron for acute CHS symptom relief in the ED, although dosing must be balanced against adverse effects [8]. Topical capsaicin targeting transient receptor potential vanilloid 1 (TRPV1) has supportive pilot and observational data and may shorten ED length of stay, but higher-quality trials remain limited [9]. Importantly, commonly used antiemetics can prolong the QT interval, and the risk is accentuated by hypokalemia, hypomagnesemia, and acidosis typical of refractory vomiting [10]. Meta-analytic and ED cohort data confirm clinically relevant, dose-related corrected QT (QTc) effects with ondansetron; intravenous haloperidol also carries the risk of QT prolongation and torsades de pointes, particularly at higher doses, prompting ECG and electrolyte surveillance in susceptible patients [11]. In our patient, ECG changes were consistent with hypokalemia (U waves and ST depression) without QT prolongation, highlighting the importance of aggressive electrolyte correction and judicious antiemetic selection.

Aromatherapy with isopropyl alcohol (IPA) has emerged as a rapid, inexpensive, non-pharmacological option for acute nausea. Multiple randomized trials in ED triage and treatment areas show faster short-term relief with IPA swab inhalation versus saline/placebo, and a pragmatic ED trial suggests reduced rescue antiemetic use [12]. Systematic reviews conclude a modest but clinically meaningful early effect with an excellent safety profile; benefits appear greatest within the first 10-30 minutes and may be leveraged while IV access is obtained or when QT‑prolonging agents are undesirable [13]. Although published reports specifically in CHS are sparse, our patient's prompt improvement after IPA inhalation, despite the failure of ondansetron, cyclizine, haloperidol, proton‑pump inhibition, and topical capsaicin, supports the early consideration of IPA as an adjunct in refractory CHS, particularly when accompanying electrolyte and acid-base abnormalities or suspected perforation make pharmacological escalation less appealing.

Sustained cannabis cessation remains the only disease-modifying therapy and is associated with the complete remission of CHS symptoms in observational cohorts and expert guidance [14]. Structured cessation counseling, linkage to substance‑use services, and clear discharge instructions about relapse risk are essential components of care [14].

For ED and inpatient teams, a practical strategy is to promptly recognize the CHS phenotype and assess for the presence of complications using chest radiography or computed tomography in cases where chest pain or subcutaneous emphysema is present. Management should prioritize fluid resuscitation and electrolyte replacement when clinical or laboratory findings indicate dehydration or electrolyte disturbances. The use of antiemetic regimens should be judicious and carefully monitored, particularly concerning the risk of QT interval prolongation. Adjunctive therapies such as topical capsaicin and early IPA inhalation should be considered for rapid and low-risk symptom relief. In addition, clinicians should initiate cessation counseling and ensure follow-up in accordance with AGA recommendations. Future prospective studies in CHS are warranted to determine optimal IPA dosing and frequency, evaluate comparative effectiveness against haloperidol or capsaicin, and examine patient-centered outcomes such as time to oral tolerance and ED length of stay.

## Conclusions

CHS should be considered in young patients with recurrent, unexplained vomiting and a history of chronic cannabis use, particularly when accompanied by compulsive hot bathing behavior. Although generally benign, CHS can rarely present with serious complications such as pneumomediastinum and pneumothorax secondary to forceful retching. Management may primarily be supportive, focusing on fluid resuscitation, electrolyte correction, and symptomatic relief, with long-term resolution dependent on cannabis cessation. Importantly, this case highlights the potential utility of inhaled isopropyl alcohol swabs as a safe, inexpensive, and rapidly effective adjunct for refractory nausea and vomiting when standard antiemetic therapy fails. The early recognition of CHS and the timely incorporation of such adjunctive therapies may reduce patient morbidity, prevent potential side effects from the pharmacological therapy, and support recovery.
